# Safe Administration of Cemiplimab to a Kidney Transplant Patient with Locally Advanced Squamous Cell Carcinoma of the Scalp

**DOI:** 10.3390/curroncol28010057

**Published:** 2021-01-19

**Authors:** Luca Paoluzzi, Thomas J Ow

**Affiliations:** 1 Department of Medicine (Oncology), Albert Einstein College of Medicine, Montefiore Medical Center, Bronx, New York, NY 10467, USA; 2 Department of Otorhinolaryngology-Head and Neck Surgery, Montefiore Medical Center, Bronx, New York, NY 10467, USA; thow@montefiore.org; 3 Department of Pathology, Montefiore Medical Center, Bronx, New York, NY 10467, USA

**Keywords:** cemiplimab, cutaneous squamous cell carcinoma, kidney transplant, checkpoint inhibitor, PD1

## Abstract

Immunotherapies directed at T-cell activation through antibodies targeting checkpoint proteins, such as programmed cell death 1 (PD1), are rapidly becoming the new standard of care in the treatment of several malignancies. Cemiplimab is a monoclonal antibody targeting PD1 that has recently emerged as a highly active treatment for locally advanced and metastatic cutaneous squamous cell carcinoma (CSCC). Patients who have received an organ transplant (OTRs) have been traditionally excluded from clinical trials with checkpoint inhibitors (CIs), given concerns for organ rejection. Renal transplant recipients (RTRs) are more likely to develop cancers than the general population, and skin cancers are among the most frequent malignancies. We report the case of a 72-year-old man with a history of a kidney transplant who presented with a rapidly growing, locally advanced squamous cell carcinoma (SCC) of the scalp that recurred within four weeks from surgical resection. The patient was able to safely receive ten cycles of cemiplimab so far with significant clinical benefit, and no issues with his kidney function, while continuing immunosuppression with low dose prednisone alone. An ongoing clinical trial (NCT04339062) is further exploring the safety of CIs in patients with metastatic CSCC who have previously received allogeneic hematopoietic stem cell transplant or a kidney transplant.

## 1. Introduction

The introduction of T-cell-targeted immunomodulators that block the immune checkpoints CTLA-4, PD1, or PDL1 represents one of the most significant advances in oncology in the last decade. Since ipilimumab was approved by the Federal Drug Administration (FDA) in 2011 for the treatment of melanoma, six additional checkpoint inhibitors have entered the therapeutic armamentarium of several solid and hematologic malignancies [1].

Cemiplimab is a recombinant IgG4 monoclonal antibody that inhibits PD1, approved by the FDA in 2018 for the treatment of patients with metastatic or locally advanced cutaneous squamous cell carcinoma (CSCC) who are not candidates for curative surgery or curative radiation. This approval was based on clinically meaningful and durable objective response rates in patients with CSCC in two clinical trials [2].

Solid organ transplant recipients have increased rates of cancer with higher incidence of CSCC [3]. In renal transplant recipients (RTRs), CSSC affects over 50% of patients, occurring earlier and behaving more aggressively than in non-transplant patients [4]. RTRs have been traditionally excluded from clinical trials with checkpoint inhibitors (Cis) due to concerns for T-cell activation and transplant rejection.

Here, we present the case of a kidney transplant patient with a rapidly-progressing, recurrent CSS of the scalp, who received the anti-PD1 antibody cemiplimab without any significant complications. To our knowledge, this is the first case of safe administration of this CI to a transplant patient on low dose prednisone alone for immunosuppression.

## 2. Case Presentation

A 72-year-old man with a history of allogeneic kidney transplantation from a living related donor ten years prior developed a highly-aggressive recurrent squamous cell carcinoma (SCC) of the scalp after undergoing multiple prior resections. The patient had several comorbidities, including hypertension, hyperlipidemia, diabetes mellitus Type 2, heart failure with left ventricular ejection fraction around 30%, severe peripheral artery disease, for which he previously underwent bypass surgery, and endarterectomy. Overall, his Eastern Cooperative Oncology Group (ECOG) performance status was 2. After kidney transplant, the patient had been on different immunosuppressive drugs including tacrolimus, mycophenolate mofetil and prednisone.

**Figure 1:**
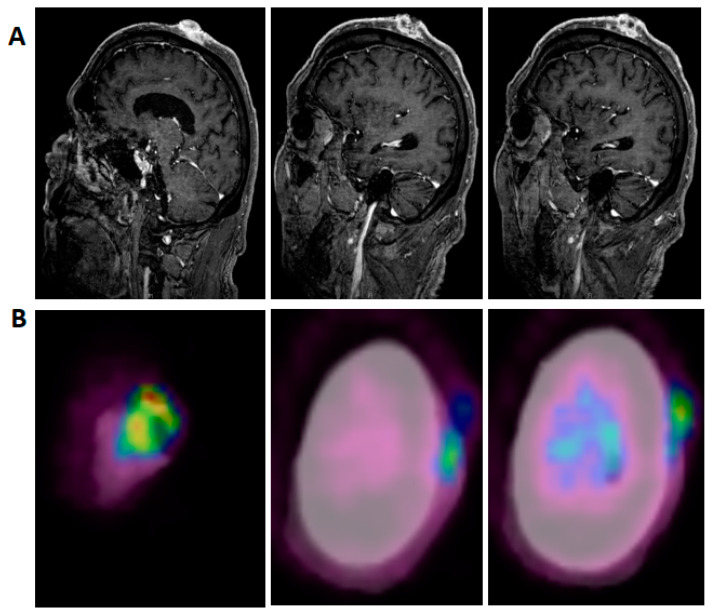
Representative pre-treatment, post-contrast, T1-weighted, sagittal MRI images (**A**) and fused PET-CT images (**B**) show disease extension predominantly from the vertex scalp to the left lateral temple region.

The patient had extensive local SCC involving the majority of his scalp, with no evidence on examination or imaging for either regional or metastatic disease. MRI of the brain and neck was done and showed irregularly enhancing scalp lesions without evidence of adjacent osseous or intracranial extension (largest lesion was 4.7 cm by 3.4 cm (Figure 1A)). PET/CT was significant for fluorodeoxyglucose (FDG) avid soft tissue masses in the left scalp, consistent with the patient’s known malignancy (standardized uptake value, SUV up to 4.7 (Figure 1B)). After tumor board discussion, decision was made for surgical resection; he underwent radical resection including full thickness resection of his scalp, and removal of the underlying external cortex of his calvarium, with placement of a collagen/glycosaminoglycan allograft to encourage granulation in preparation to receive a split thickness skin graft. Pathology showed a moderately differentiated SCC with negative margins (the closest, deep margin was 2 mm); size was 8.3 cm, depth of invasion was 1.1 cm; perineural invasion was identified. Within four weeks since resection, the patient developed extensive dermal metastasis surrounding the resection bed, which on biopsy were confirmed to be poorly differentiated CSCC.

Mycophenolate mofetil was stopped, and the patient continued immunosuppression with only low dose prednisone (5 mg daily). At that point, the patient was willing to consider systemic treatment, including a CI, despite the lack of safety data in transplant patients and the theoretical risks of kidney rejection.

After further multidisciplinary discussion with the surgical and transplant teams, given the fact he was not a candidate for any aggressive regimen due to his performance status, decision was made for immunotherapy with cemiplimab 350 mg IV every 3 weeks. The patient’s baseline creatinine and glomerular filtration rate (GFR) before starting cemiplimab were 1.5 and 46, respectively.

**Figure 2:**
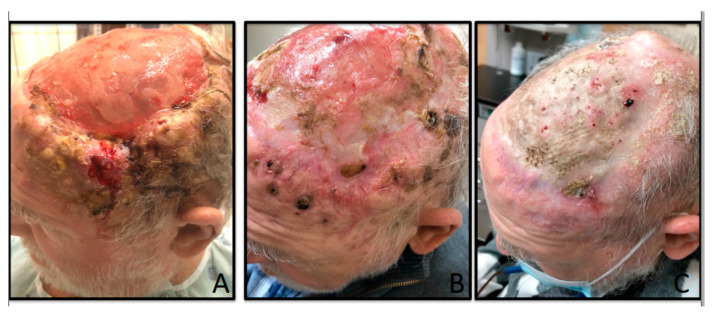
(**A**) Multiple nodules consistent with cutaneous squamous cell carcinoma on the left temporal area before starting cemiplimab; (**B**) tumor regression after three cycles of cemiplimab; (**C**) response after a total of 10 cycles of cemiplimab.

After the first administration of cemiplimab, the patient started noticing decrease in size of several scalp lesions, with additional benefit noticed after further treatment (Figure 2A,B); creatinine and GFR were 1.08 and 67, respectively, after a total of six cycles. At that point, cemiplimab was stopped because the patient had to undergo vascular surgeries of the lower extremities and split thickness graft to the scalp. Surgeries were complicated by wound infection from polymicrobial flora that required protracted antibiotic therapy. Cemiplimab was on hold for about five months with some progression of disease in the scalp, but it was ultimately resumed, with ongoing clinical benefit after a total of 10 cycles (Figure 2C). Overall, the patient has tolerated this drug very well so far without any significant issues. Creatinine and GFR before cycle 10 were 0.85 and 87, respectively (Table 1). Spearman’s rank correlation (Graphpad Prism software, San Diego, CA, USA. www.graphpad.com) and t-testing with equal variance were used to analyze the changes in creatinine/GFR through the patient’s 10 cycles of therapy. Over time, creatinine level decreased and GFR improved during treatment with a strong positive correlation between cycle of treatment and kidney function (0.837, *p* = 0.003); creatinine/GFR levels between the first three cycles of treatment compared to the last three cycles were significantly improved (*p* = 0.02).

## 3. Discussion

Patients who undergo a renal transplant are approximately three times more likely to develop different type of cancers than the general population [5]. Risk factors specific to the transplant population include the type, extent, and duration of immunosuppression. CSCC is the most common skin cancer in renal transplant patients. In comparison with SCC in immunocompetent patients, SCC in organ transplant recipients is more likely to manifest as aggressive disease [6,7,8]. While the modulation of immunotherapy plays an important role in the management of these patients, additional cancer-directed treatments are often necessary to achieve appropriate tumor control. Systemic therapies based on platinum agents, capecitabine, or cetuximab can help achieving tumor responses, but such responses are generally short-lived; additionally, these drugs can be problematic in patients with a suboptimal performance status due to multiple comorbidities.

PD1 is a receptor of the immunoglobulin superfamily expressed on T cells, B cells, and NK cells; it negatively regulates the T-cell antigen receptor signaling by interacting with specific ligands expressed in multiple tumor types, including tumor cells. While the expression of the ligand PD-L1 can serve as a potent mechanism for potentially immunogenic tumors to escape from host immune responses, blockade of the PD1–PD–L1 interaction through specific monoclonal antibodies has shown to provide effective and durable antitumor effects in several malignancies [9,10,11].

In non-transplant patients with locally advanced or metastatic SCC that is not amenable to treatment with surgery or radiation therapy, the anti-PD1 monoclonal antibodies cemiplimab and pembrolizumab have recently shown to be potentially more active and relatively less toxic than traditional chemotherapy. Unfortunately, the efficacy and safety of these agents in OTRs is unknown at this time, given the exclusion of such patients from clinical trials. Furthermore, case reports and small retrospective series have described transplant rejection in at least 40% of subjects who received CIs after undergoing a kidney transplant [12,13,14,15,16,17].

In particular, De Bruyn et al. [13] reported 48 cases of RTRs and liver recipients who had received CIs; of the 29 RTRs, 13 (45%) experienced rejection. From a cancer standpoint, 7 out of 13 patients who had a rejection (53%) had a favorable outcome.

In another review of 39 OTRs treated with CIs, rejections rates were highest in the 23 RTR (48%), compared to liver (36%) and cardiac (20%) transplant patients [14].

Manohar et al. [15] recently conducted a literature search and reviewed 27 studies that reported outcomes of 44 RTRs who had received treatment with CIs; 11% of these patients presented with metastatic CSCC, 68% had melanoma, while the remaining patients had either lung, Merkel cell, urothelial, or duodenal carcinomas. Most patients received a CI as a single agent: nivolumab in 34% cases, pembrolizumab in 25%, ipilimumab in 20%, and avelumab in one patient; the remaining cases received two different CIs sequentially. Overall, 18 patients (41%) were reported to have acute rejection of the renal allograft with a median time of 24 days; eventually, 15 out of these 18 patients had allograft failure (83%), and 8 patients died. Reported types of acute allograft rejection were cellular rejection (33%), mixed cellular and antibody-mediated rejection (17%), and unspecified type (50%). Baseline immunosuppressive regimen data were available for 31 patients: 48% of them were on a triple regimen with a calcineurin inhibitor, such as tacrolimus or cyclosporine, mycophenolate mofetil, and low-dose steroids.

In another large systematic review, 83 OTRs were treated with CIs (about two-thirds received anti-PD1/PDL1 therapy, 16% anti-CTLA-4, 11% combination therapy) [16]. This study included 53 RTRs; allograft rejection occurred in 40% of patients, leading to end-stage organ failure in 71% of cases. Outcomes were similar across organs and immunotherapy regimens. Patients on no other immunosuppressive treatment besides corticosteroids at initiation of CIs had a higher risk of rejection. RTR was the only group where there was similar mortality in patients with and without rejection, suggesting that CIs may be a better option in these patients where hemodialysis is a life-saving alternative in case of rejection [16,17].

Ali et al. [18] recently reported successful administration of cemiplimab to an 81-year-old patient with advanced CSCC on sirolimus and prednisone for immunosuppression after renal transplantation. Sirolimus is a known inhibitor of the mechanistic target of rapamycin (mTOR). Interestingly, mTOR inhibitors have been shown to reduce the growth and proliferation of tumor cells in vitro and in vivo mouse models [19]. In a mixed-organ cohort of OTRs, patients taking sirolimus after developing post-transplant cancer showed lower risk of developing skin cancer [20]. The immunosuppression provided by sirolimus to the patient above may have mitigated the rejection effect from cemiplimab, while boosting the anti-cancer response.

The improvement in kidney function observed in our patient over time is difficult to interpret; it may be related to closer patient’s monitoring with frequent assessments of his hydration status in the context of multiple office and hospital visits.

## 4. Summary

To our knowledge, this is the first report of safe administration of the anti-PD1 monoclonal antibody cemiplimab in a kidney transplant patient with recurrent, locally advanced SCC of the scalp treated with prednisone alone for immunosuppression. The ongoing phase 1/2 CONTACT clinical trial is exploring the safety and effectiveness of cemiplimab in subjects with advanced SCC who have previously received an allogeneic hematopoietic stem cell transplant or kidney transplant (NCT04339062); all patients on this trial are required to be on immunosuppression with prednisone and either sirolimus or everolimus.

## Figures and Tables

**Table 1:** Changes in creatinine and glomerular filtration rate (GFR) during treatment with cemiplimab (before each cycle).

Cemiplimab	Creatinine (Ref Range < l.5 mg/dL)	GFR (Ref Range > 60 mL/min/BSA)
C#1	1.2	60
C#2	1.1	66
C#3	1.0	74
C#4	1.2	60
C#5	1.2	60
C#6	1.0	74
C#7	0.97	78
C#8	0.88	86
C#9	0.91	84
C#10	0.85	87

Ref = reference; C = cycle.
